# Efficacy and safety of rituximab in primary IgA nephropathy: a retrospective study

**DOI:** 10.1007/s10157-024-02617-0

**Published:** 2024-12-27

**Authors:** Jingzhen Li, Zhenyu Nie, Guofu Li, Beiyan Bao

**Affiliations:** Department of Nephrology, Ningbo Yinzhou Second Hospital, No. 998, North Qianhe Road, Yinzhou District, Ningbo City, 315000 Zhejiang Province China

**Keywords:** IgA nephropathy, Proteinuria, Renal function, Retrospective study, Rituximab

## Abstract

**Purpose:**

The study aimed to evaluate the efficacy and safety of rituximab (RTX) in primary IgA nephropathy (IgAN).

**Methods:**

A retrospective review was conducted on the medical records of 22 patients diagnosed with primary IgAN who received RTX treatment. The clinical data, including blood tests, urine examinations and estimated glomerular filtration rate (eGFR), were analyzed at four time point: baseline, 3 months, 6 months and 12 months. Adverse events were also recorded.

**Results:**

Our study included 9 male and 13 female participants. The level of serum albumin significantly increased after three months with RTX applied (*P* < 0.01). Furthermore, we observed a significant reduction in microalbuminuria and urine albumin-to-creatinine ratio at twelve months (*P* < 0.01). However, there was no change in serum creatinine (*P* = 0.08), urinary red blood cell (*P* = 0.11) or eGFR (*P* = 0.09) during the course of one year. Two cases achieved complete remission, while eleven cases experienced partial remission, resulting in an overall remission rate of 50.0%. During the treatment period, three patients developed infections and two patients encountered infusion-related adverse reactions.

**Conclusion:**

In our retrospective study, RTX demonstrated a significant improvement in serum albumin levels and a reduction in proteinuria among primary IgAN patients. Although no statistically significant difference was observed in terms of renal function, there was an observable trend towards improvement. Therefore, we propose that RTX may be an alternative treatment option for primary IgAN patients who cannot tolerate glucocorticoids or immunosuppressants.

## Introduction

IgA nephropathy (IgAN) is considered the most common glomerular disease in the world, especially in China. Although the specific mechanism of IgAN is complex, it is clear that galactose-deficient IgA1 (Gd-IgA1) plays a critical role. Elevated levels of Gd-IgA1 are found in the circulation of most IgAN patients, which is related to IgG or IgA autoantibodies binding to Gd-IgA1 and the subsequent formation of immune complexes [1]. These immune complex deposits lead to mesangial cell activation, matrix production, proinflammatory cytokine release and endocapillary influx of inflammatory cells, and all these processes ultimately lead to sclerosis [2]. IgAN patients with decreased renal function and proteinuria > 1 g/24 h have a poor prognosis, with up to 50% progressing to end-stage renal disease (ESRD) within ten years [3]. As a standard practice, angiotensin-converting enzyme inhibitors (ACEIs) or angiotensin-receptor blockers (ARBs) are used to treat mild to moderate proteinuria. However, treating patients with IgAN at high risk of progressive chronic kidney disease is still controversial. Several studies have demonstrated that glucocorticoids (GCs) prevent deterioration of renal function without remarkable side effects [4, 5]. Nevertheless, Rauen et al. [6] found there was no significant difference in the outcomes between patients receiving supportive care plus immunosuppression and those receiving supportive care alone for IgAN. Due to the risks of glucocorticoid-related toxicity, there remains an urgent demand to find better and safer treatments for IgAN.

Rituximab (RTX) is an anti-CD20 monoclonal antibody functioning as a B-cell inhibitor. It has been proven to be an effective therapy in many immune-mediated kidney diseases over time, such as membranous nephropathy (MN) [7], systemic lupus erythematosus (SLE) [8], cryoglobulinemia [9] and ANCA-associated vasculitis [10]. The potential efficacy of RTX as a therapeutic intervention could be attributed to its ability to target autoimmune mechanisms, which are believed to underlie the pathogenesis of IgAN. Up to now, the effectiveness of RTX remains to be ascertained. Several reports have discovered the benefit of RTX in IgAN or IgA vasculitis [11–13]. However, a randomized, controlled trial in patients at risk of progressive IgAN reported that RTX could not improve the estimated glomerular filtration rate (eGFR) or proteinuria and was associated with more adverse events [14]. Data regarding the effectiveness and safety of RTX in IgAN are limited.

Therefore, our study aimed to retrospectively analyse our daily practice experience with RTX in IgAN by evaluating its effectiveness and safety.

## Materials and method

### Subject

The study included all patients with IgAN treated with RTX between June 2016 and June 2022 at the Ningbo Yinzhou Second Hospital. The inclusion criteria were patients with (i) age between 18 and 80 years, (ii) renal biopsy confirmed IgAN, (iii) RTX had not been used previously, (iv) eGFR > 30 ml/(min·1.73 m^2^), (v) follow-up for at least 12 months. The exclusion criteria were patients with (i) IgAN in combination with other glomerulonephritis, (ii) IgA vasculitis, SLE, viral hepatitis, liver cirrhosis and other secondary IgAN, (iii) missing clinical and laboratory data, (iv) administered immunosuppressants in the meantime, (v) the intial treatment RTX was incompleted.

### Research methods

All patients in this study recorded their previous treatment regimens, renal pathology, and the risk of kidney progression at 1 and 5 years using the International IgAN Prediction Tool. The baseline was defined as the day the initial RTX injection was administered. The clinical data of the patients, which included blood tests, urine examinations and eGFR, were analyzed across four time points: baseline, 3 months, 6 months and 12 months following the commencement of RTX, respectively. This analysis was conducted over a period of one year to observe any changes or trends. Two treatment protocols were administered in the context of RTX. The patients were treated with RTX 375 mg/m^2^ weekly for a duration of four weeks, or 1 g on days 1 and 15. At the sixth month, if the CD19 cell count exceeds 5/ul, it is generally recommended to consider additional administration of 1 g RTX. To minimize reactions to the RTX infusions, desloratadine citrate disodium (8.8 mg) was administered orally and dexamethasone (5 mg) was administered intravenously at least 30 min before each infusion.

### Observation indicators

This assessment included the measurement of routine blood tests, the count of CD19, urine examinations and adverse events. The eGFR was calculated by the CKD Epidemiology Collaboration (CKD-EPI) 2009 formula, which takes into account the laboratory-measured serum creatinine (Scr) concentration. The duration of the follow-up period was 12 months.

The primary outcome measures were the change in albumin (Alb), Scr, urinary red blood cell (URBC), microalbuminuria (MAU), urine albumin-to-creatinine ratio (UACR) and eGFR at four different time points. The secondary outcome was safety-related, recording overall adverse events such as infections and infusion-related reactions. Complete remission was defined as 24-h urine protein quantification < 0.3 g/d, Alb > 30 g/L and normal renal function. Partial remission was defined as 24 h urine protein quantification range from 0.3 to 3.5 g/d, a decrease of 50% or more from baseline, Alb ≥ 30 g/L and stable renal function. These indicators were collected via the hospital’s inpatient electronic medical record system, the outpatient system and the Ningbo electronic medical record retrieval system.

### Statistical analyses

The Shapiro–Wilk test was used to test for normal distributions. Comparing means of non-normally distributed data was done with the Friedman test. Post hoc analysis was also done using Friedman’s 2-way analysis of variance (ANOVA) by ranks. A one-way ANOVA was conducted to assess the time-response effect of eGFR after administering RTX. The sphericity test was performed before repeated-measures ANOVA. If the data did not satisfy the sphericity test, the Greenhouse–Geisser method was used for correction. Descriptive statistical analyses were undertaken with range values, including the mean and median. Statistical significance was defined by a *P*-value < 0.05. Statistical analyses were performed in IBM SPSS Statistics Version 26.

## Results

### Baseline characteristics

A total of 22 patients with IgAN were enrolled in this study. The patient characteristics at baseline are listed in Table 1. The mean age of patients who initially received RTX treatment was 47.95 ± 16.97 years old. This study included 9 males and 13 females. Refractory nephropathy was observed in 10 out of the 22 patients who received treatment with GCs alone or in combination with immunosuppressants, such as calcineurin inhibitors, mycophenolate mofetil, tripterygium glycosides, leflunomide, and cyclophosphamide. The other 12 patients chose RTX due to concerns about side effects of GCs. Before receiving RTX, 20 patients were provided with ACEIs or ARBs supportive treatment. Two cases were excluded, one due to hypotension and the other due to a progressive increase in Scr. The baseline median (interquartile range (IQR)) proteinuria of all subjects was 3.55 (1.43, 6.08) g/d. There were only 4 patients with levels of proteinuria < 1 g/d. Among these 22 patients, 20 were diagnosed with chronic nephritis syndrome, while the remaining 2 were diagnosed with nephrotic syndrome. Furthermore, it was observed that within one year, a total of 4 individuals progressed to chronic kidney failure.

**Table 1:** The baseline characteristics of 22 patients with primary IgA nephropathy

Baseline characteristics (*n* = 22)	
Mean age, years	47.95 ± 16.97
Gender (Male, Female)	9, 13
Hypertension	11
Diabetes	6
SBP (mmHg)	113.00 ± 9.80
DBP (mmHg)	72.86 ± 7.58
Hemoglobin (g/dL)	12.10 ± 0.31
Alb (g/L)	34.50 (30.75, 36.00)
Scr (mg/dL)	1.16 (0.86, 1.51)
URBC (/ul)	56.00 (11.75, 270.25)
MAU (mg/L)	2100.00 (870.75, 3664.75)
UACR (mg/mmol)	180.50 (106.75, 397.75)
eGFR (ml/(min·1.73 m^2^))	64.91 ± 26.93
24-h urine protein quantification (g/d)	3.55 (1.43, 6.08)
*RTX treatment schedule*	
375 mg/m^2^ *4	8
1 g*2	14
Additional1g RTX at 6 months	13
Initial treatment	12
Refractory	10

*SBP* systolic blood pressure, *DBP* diastolic blood pressure, *Alb* albumin, *Scr* serum creatinine, *URBC* urinary red blood cell, *MAU* microalbuminuria, *UACR* urine albumin-to-creatinine ratio, *eGFR* estimated glomerular filtration rate, *GCs* glucocorticoids, *ISDs* immunosuppressive drugs, *RTX* rituximab

### Efficacy and prognosis

**Fig. 1:**
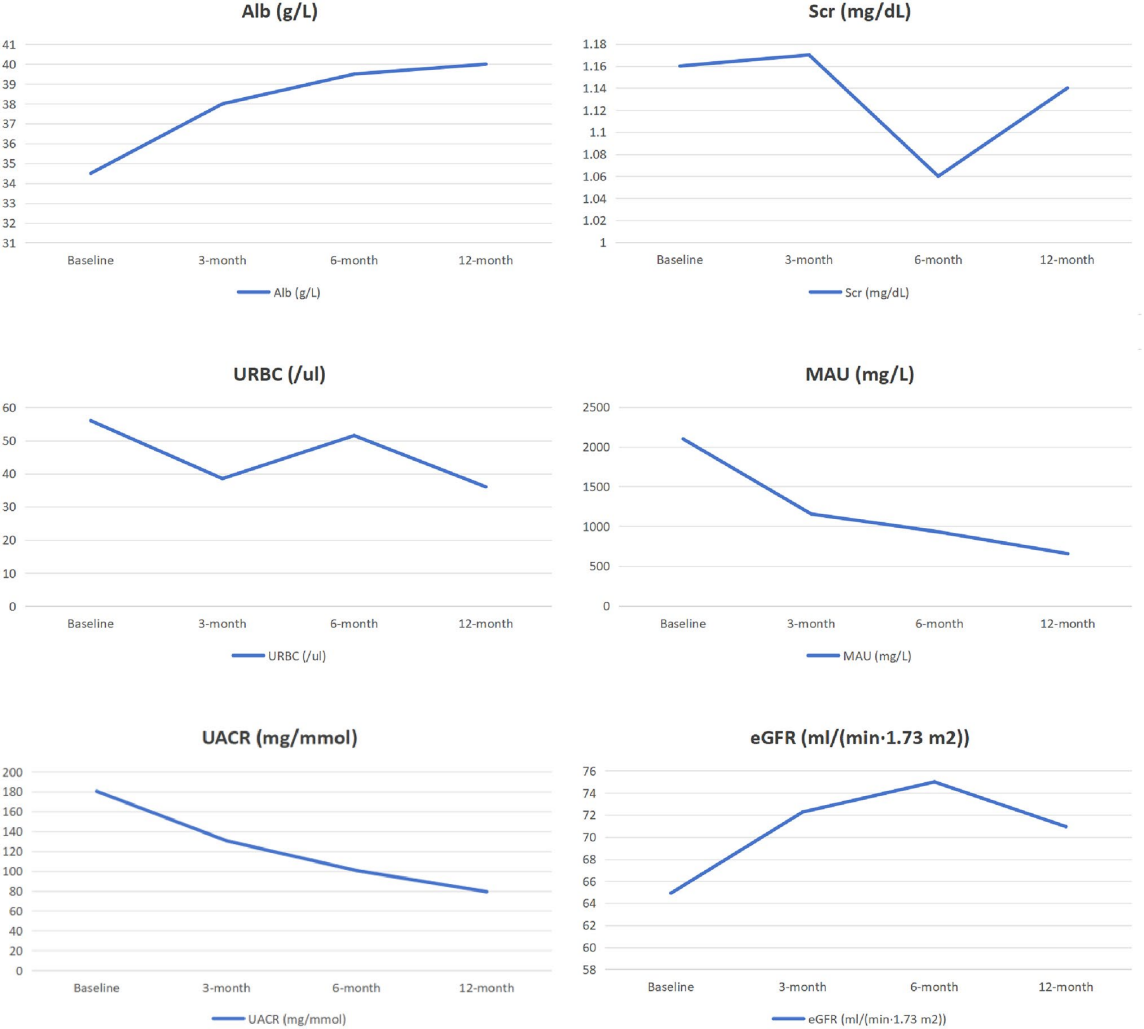
Changes in indicators within one year following rituximab treatment in primary IgA nephropathy

The Shapiro–Wilk test of normality has shown that *P* < 0.05 for Alb, Scr, URBC, MAU and UACR, suggests that these data were not normally distributed. The eGFR was normally distributed *P* > 0.05. Variations in indicators in primary IgAN patients treated with RTX are shown in Table 2 and Fig. 1. The median (IQR) of Alb for each time point was as follows: baseline was 34.50 (30.75, 36.00) g/L, 3 months was 38.00 (34.75, 42.00) g/L, 6 months was 39.50 (36.75, 41.25) g/L and 12 months was 40.00 (37.00, 42.35) g/L. There was a statistically significant change in the level of Alb after the application of RTX (Χ^2^ = 28.56, *P* < 0.01). A post hoc pairwise analysis was conducted with a Bonferroni correction applied. There was a notable difference between baseline measurements and those taken at 3 months (Z = – 3.68, *P* < 0.01), 6 months (Z = – 4.09, *P* < 0.01), and 12 months (Z = – 4.84, *P* < 0.01). The results suggested that Alb was significantly improved after treatment with RTX for three months, but there was no further improvement at subsequent times. The median (IQR) of Scr at baseline was 1.16 (0.86, 1.51) mg/dL, at 3 months was 1.17 (0.82, 1.38) mg/dL, at 6 months was 1.06 (0.75, 1.52) mg/dL, and at 12 months was 1.14 (0.73, 1.50) mg/dL. The median (IQR) of URBC at each different point-time was as follows: 56.00 (11.75, 270.25) /ul, 38.50 (8.50, 210.25) /ul, 51.50 (13.75, 135.00) /ul and 36.00 (3.00, 122.50) /ul, respectively. Over the course of one year, there were no significant statistical differences in Scr and URBC compared to baseline (*P* > 0.05).

**Table 2:** Changes in indicators within one year following rituximab treatment in primary IgA nephropathy

Indicators	Baseline	3-month	6-month	12-month	Χ^2^/F	P
Alb (g/L)	34.50 (30.75, 36.00)	38.00 (34.75, 42.00)^a^	39.50 (36.75, 41.25)^a^	40.00 (37.00, 42.35)^a^	Χ^2^ = 28.56	*p* < 0.01
Scr (mg/dL)	1.16 (0.86, 1.51)	1.17 (0.82, 1.38)	1.06 (0.75, 1.52)	1.14 (0.73, 1.50)	Χ^2^ = 6.69	*p* = 0.08
URBC (/ul)	56.00 (11.75, 270.25)	38.50 (8.50, 210.25)	51.50 (13.75, 135.00)	36.00 (3.00, 122.50)	Χ^2^ = 6.01	*p* = 0.11
MAU (mg/L)	2100.00 (870.75, 3664.75)	1154.00 (409.25, 2311.00)	929.50 (221.75, 1713.00)	658.00 (195.50, 1054.25)^ab^	Χ^2^ = 19.15	*p* < 0.01
UACR (mg/mmol)	180.50 (106.75, 397.75)	130.50 (31.25, 277.00)	100.50 (46.08, 174.00)	79.25 (21.85, 156.25)^a^	Χ^2^ = 11.84	*p* < 0.01
eGFR (ml/(min·1.73 m^2^))	64.91 ± 26.93	72.27 ± 31.50	75.00 ± 32.64	70.94 ± 33.76	F = 2.84	*p* = 0.09

*Alb* albumin, *Scr* serum creatinine, *URBC* urinary red blood cell, *MAU* microalbuminuria, *UACR* urine albumin-to-creatinine ratio, *eGFR* estimated glomerular filtration rate, ^a^There was a significant statistical difference from the baseline, ^b^There was a significant statistical difference from 3-month

The median (IQR) of MAU at baseline was 2100.00 (870.75, 3664.75) mg/L, at 3 months was 1154.00 (409.25, 2311.00) mg/L, at 6 months was 1154.00 (409.25, 2311.00) mg/L and at 12 months was 658.00 (195.50, 1054.25) mg/L. The median (IQR) of UACR for each point-time was 180.50 (106.75, 397.75) mg/mmol, 130.50 (31.25, 277.00) mg/mmol, 100.50(46.08, 174.00) mg/mmol and 79.25(21.85, 156.25) mg/mmol, respectively. Compared with the baseline, this study showed a significant decrease in MAU (Χ^2^ = 19.15, *P* < 0.01) and UACR (Χ^2^ = 11.84, *P* < 0.01) after RTX treatment. A post hoc pairwise analysis was conducted with a Bonferroni correction applied. MAU showed a significant difference between baseline and 12 months (Z = 4.20, *P* < 0.01), 3 months and 12 months (Z = 2.92, *P* = 0.02). MACR showed a significant difference between baseline and 12 months (Z = 3.386, *P* < 0.01). These findings indicated a significant reduction in MAU and MACR following the administration of RTX for a duration of one year.

The mean ± standard deviation of eGFR for each time point was as follows: baseline was 64.91 ± 26.93 ml/(min·1.73 m^2^), 3 months was 72.27 ± 31.50 ml/(min·1.73 m^2^), 6 months was 75.00 ± 32.64 ml/(min·1.73 m^2^) and 12 months was 70.94 ± 33.76 ml/(min·1.73 m^2^). The data of eGFR did not satisfy the sphericity test, so the Greenhouse–Geisser method was used for correction. Repeated measures ANOVA suggested no statistically significant differences in eGFR between each time point (F = 2.84, *P* = 0.09).

Two cases had complete remission, eleven had partial remission, and the total remission rate was 50.0%. After a two-week treatment with RTX, all subjects demonstrated a CD19 B cell count below 5/ul. The previous treatment procedures, renal pathology grading, and efficacy assessment are presented in Table 3.

**Table 3:** The previous treatment procedures, grading of renal pathology,and efficacy assessment in 22 IgAN patients

No	Age	Gender	Time of treatment	Prior treatment	Pathology (Lee’s)	MEST-C	1 and 5 years’ risk of kidney progression	UTP > 1 g/d	GCs	Efficacy evaluation
1	80	Male	3 M	No	IV	M1E0S1C1T1	0.92%, 33.46%	Yes	No	Invalid
2	39	Female	1Y	RAAS	IV	M1E1S1C2T0	0.27%, 11.11%	No	No	Invalid
3	47	Male	16Y	RAAS + GCs/Tac/CTX	III	No data	No data	Yes	No	Invalid
4	62	Female	1Y	RAAS	III	M1E1S0C2T0	1.52%, 49.23%	Yes	Yes	CR
5	58	Female	20Y	RAAS + GCs/Tac/MMF	III	M1E1S0C0T0	3.43%, 23.52%	Yes	No	CR
6	20	Female	3Y	RAAS	III	M1E1S1C1T0	0.17%, 7.38%	No	No	PR
7	34	Female	6 M	RAAS	III	M1E1S1C1T0	0.29%, 12.12%	Yes	No	PR
8	59	Male	2 M	No	III	M1E1S1C0T1	3.80%, 82.05%	Yes	Yes	PR
9	32	Female	8Y	RAAS	II	No data	No data	No	No	Invalid
10	50	Female	11Y	RAAS + GCs	IV	M1E1S1C1T0	0.87%, 32.22%	Yes	No	Invalid
11	50	Female	1Y	RAAS	III–IV	M1E1S1C2T0	0.21%, 8.98%	No	No	PR
12	50	Female	8Y	RAAS + GCs + TGT/AZA	III	M1E1S1C0T0	1.09%,36.63%	Yes	No	Invalid
13	66	Male	6Y	RAAS + TGT	III	No data	No data	Yes	No	Invalid
14	32	Female	2Y	RAAS	III	M1E1S0C2T0	0.59%, 23.11%	Yes	No	PR
15	78	Male	5Y	RAAS + TGT	III	M1E1S1C0T0	0.36%, 14.78%	Yes	No	PR
16	70	Female	10Y	RAAS + GCs + TGT/LEF	III	No data	No data	Yes	No	PR
17	36	Male	2Y	RAAS + GCs + Tac	III	M1E1S1C1T0	5.88%, 37.20%	Yes	No	PR
18	53	Male	2Y	RAAS	III	M1E1S1C1T0	14.52%, 70.02%	Yes	No	PR
19	26	Male	1 M	No	IV	M1E1S1C1T1	2.66%, 69.75%	Yes	Yes	Invalid
20	50	Male	4Y	RAAS + GCs + CTX	III	M1E0S1C0T0	2.66%, 18.69%	Yes	No	Invalid
21	36	Female	8Y	RAAS + TGT	III	M1E1S0T0	7.52%, 45.16%	Yes	Yes	PR
22	36	Female	3Y	RAAS	II–III	M1E1S1C0T0	3.98%, 26.79%	Yes	Yes	PR

*No* Number, *UTP* urine total protein, *GCs* glucocorticoids, *M* moths, *Y* years, *RAAS* renin–angiotensin–aldosterone system, *Tac* tacrolimus, *CTX* cyclophosphamide, *MMF* mycophenolate mofetil, *TGT* tripterygium glycosides tablets, *LEF* leflunomide, *CR* complete remission, *PR* partal remission

*Alb* albumin, *Scr* serum creatinine, *URBC* urinary red blood cell, *MAU* microalbuminuria, *UACR* urine albumin-to-creatinine ratio, *eGFR* estimated glomerular filtration rate

### Adverse

There was no serious adverse event in our retrospective study. The majority of adverse events were infectious (*n* = 3). One case was a pulmonary cryptococcus infection and the other two were urinary tract infections. During the infusion procedure, a total of two patients (9.09%) experienced adverse reactions that were related to the infusion. These responses included the development of a rash and a flushed face. It is important to note that these symptoms were promptly alleviated through the administration of appropriate symptomatic medication.

## Discussion

Currently, the pathogenesis of IgAN is partially clear but is believed to involve a four-hit theory [1]. (i) The key factor is increased Gd-IgA1, which muco-associated lymphoid tissue produces. Mucosal infection promotes the activation of B cells through T-cell-dependent and non-T-cell-dependent pathways. Serum B cells activation factor and a proliferation-inducing ligand both promote the conversion of B cells into plasma cells that produce Gd-IgA1. Therefore, the amount of Gd-IgA1 was secreted into the circulation. (ii) anti–Gd–IgA1 antibodies are produced. (iii) Gd-IgA1 forms an immune complex in the systemic circulation with the antibody. (iv) The deposition of these complexes in the glomerulus leads to complement activation and inflammation in the kidney.

Up to date, the IgAN first-line treatment regimen includes RAS blockers, GCs and immunosuppressants. The KDIGO guidelines recommend that these patients with eGFR > 50 mL/min/1.73m^2^ could be considered to receive the GCs alone or combined with immunosuppressive drugs [15]. IgAN patients had different heterogeneities in terms of clinical manifestation and risk of progression [16]. The efficacy and safety of first-line therapy remain controversial. Immunosuppression in IgAN has become confusing in the past few years. In the STOP-IgAN trial, the addition of immunosuppressive therapy in patients with high-risk IgAN did not significantly improve the eGFR, and more adverse effects were observed [17]. Even after a decade of follow-up, no discernible benefits have been observed in IgAN patients undergoing immunosuppressive therapy [6]. The administration of immunosuppressive therapy was found to be ineffective in preventing the deterioration of renal function and was associated with significant adverse events [18]. Immunosuppressive therapy has demonstrated significant efficacy in reducing proteinuria, stabilizing kidney function, improving anemia, and mitigating acute kidney injury in the short term for IgAN patients with partial crescent [19]. As is well known, IgAN is well acknowledged as a chronic progressive disease. Approximately 25% of IgAN patients developed ESRD at 10-year follow-up [11]. Based on the four-hit theory new studies of treatments for IgAN include targeting pathogenic IgA1 production and complement activation. RTX might reduce the formation of immune complexes related to IgA1 and limit IgAN activity sequentially [20]. Wiercinski et al. [21] study showed an increased CD19 in children with IgAN and Schonlein-Henoch purpura. RTX may be a plausible therapy for patients with IgAN. However, there are scarce studies on RTX in primary IgAN, and its effectiveness remains a subject of much debate. Lundberg et al. [12] reported two cases of primary crescentic IgAN. Both individuals exhibited a reduction in proteinuria after the administration of RTX. Lafayette et al. [14] conducted an RCT that included 34 IgAN patients with proteinuria > 1 g/d. The results revealed that RTX could not effectively reduce proteinuria or improve eGFR. Sugiura et al. [22] shown that RTX does not exhibit efficacy as a therapy for IgAN. However, the therapeutic efficacy of RTX exhibits variability in cases of secondary IgAN. RTX has demonstrated efficacy and safety in inducing and maintaining long-term remission in patients with severe IgA vasculitis (IgAV) accompanied by aggressive renal involvement [23]. Another small cohort study supported the potential efficacy of the anti-CD20 monoclonal antibody RTX in adults with IgAV [24]. Our study showed partial or complete remission with RTX in 50.0% of IgAN. RTX is not a routine first-line treatment. IgAN was treated with RTX in our study for relapsing or refractory disease, or they had contraindications to GCs or immunosuppressive therapy. Although we observed a trend towards eGFR improvement after RTX treatment, there was no statistically significant improvement in renal function across the four distinct time periods examined.

Hematuria is a prevalent manifestation in IgAN. A meta-analysis revealed a potential association between hematuria and the progression of renal disease in IgAN patients. However, IgAN patients who present with microscopic hematuria may still achieve favorable long-term outcomes. In our study, a reduction in proteinuria was observed but there was no significant change in hematuria. A systematic review indicated that RTX could effectively reduce both hematuria and proteinuria in IgAV [25]. A reduction in proteinuria levels can be observed within several weeks to months, while hematuria improvement may take several years. Sevillano et al. [26] reported that the remission of hematuria occurred with a median time of 5.98 ± 5.92 years. These results further substantiate the notion that hematuria improvement necessitates a prolonged period.The novel oral targeted-release budesonide formulation, Nefecon, effectively inhibits the formation of Gd-IgA1 and has demonstrated a significant improvement in hematuria in a recent study [27]. Lafayette et al. [14] found that the levels of Gd-IgA1 or antibodies against Gd-IgA1 did not change after RTX treatment. This may explain the lack of improvement in hematuria following rituximab treatment. Currently, there is a lack of studies regarding the treatment of hematuria resulting from IgAN with RTX, and further investigations are warranted to validate.

Previous studies have shown that the administration of RTX is typically well received by patients without significant adverse effects [28, 29]. It is much safer than conventional treatments such as GCs and immunosuppressants. The main adverse events of RTX are infection, infusion reaction, delayed neutropenia and hypogammaglobulinemia [30]. Our study conducted observed a total of five adverse occurrences, which accounted for 22.7% of the whole sample. Fortunately, none of these incidents resulted in serious effects. There were three instances of infection and two instances of adverse events due to infusion. A reasonable increase in infection risk after RTX became apparent was observed. In a retrospective study of 370 patients with various autoimmune diseases, the rate of severe infection events was 5.3 per 100 patient-years [31].

This study may have the following limitations: (i) This retrospective observational study had a limited sample size, potentially introducing bias into the results. (ii) This study was conducted at a single center, which could result in selection bias. (iii) No additional investigation on the level of Gd-IgA1.

Although our study design has certain limitations and is not as robust as randomized controlled trials (RCTs), we still believe that this study holds significant importance. In our center, most supportive care primarily applies to IgAN patients with minimal urinary protein and no risk of progressive kidney disease. However, in this study, all 22 IgAN patients received comprehensive supportive care; among them, 21 patients were at a high risk of experiencing a decline in kidney function. Due to the presence of selection bias, it was not feasible to compare the RTX groups with the supportive care group directly. A suitable research design would involve comparing the efficacy of RTX with glucocorticoids. Our team has previously collected partial data on these two patient groups, which indicated that RTX exhibited comparatively lower effectiveness than glucocorticoids. The primary objective of this retrospective study is to examine the efficacy of RTX in individuals who are unable to tolerate the side effects of glucocorticoids or have contraindications for their use, as well as those with refractory kidney disease.

## Conclusions

To our knowledge, it was the largest retrospective study of primary IgAN treated with RTX. In our retrospective study, RTX demonstrated a significant improvement in serum albumin levels and a reduction in proteinuria among primary IgAN patients. Although no statistically significant difference was observed in terms of renal function, there was an observable trend towards improvement. Therefore, we propose that RTX may be an alternative treatment option for primary IgAN patients who cannot tolerate glucocorticoids or immunosuppressants.

## Data Availability

The datasets used and/or analysed during the current study available from the corresponding author on reasonable request.
